# siRNA against TSG101 reduces proliferation and induces G0/G1 arrest in renal cell carcinoma – involvement of c-myc, cyclin E1, and CDK2

**DOI:** 10.1186/s11658-018-0124-y

**Published:** 2019-01-15

**Authors:** Chen Xu, Junhua Zheng

**Affiliations:** Department of Urology, Tenth People’s Hospital of Tongji University, Yanchang Road 301, Shanghai, 200072 China

**Keywords:** Renal cell carcinoma, TSG101, Cell proliferation, Cell cycle

## Abstract

**Objective:**

The tumor susceptibility gene 101 (TSG101) is closely associated with various tumor types, but its role in the pathogenesis of renal cell carcinoma (RCC) is still unknown. This study used RNA interference to silence the expression of TSG101 in RCC cell lines and explore the role of TSG101 in RCC.

**Methods:**

Immunohistochemistry and western blot were performed to detect the expression of TSG101 in 15 paired renal tumor samples. A small interfering RNA (siRNA) targeting TSG101 was transfected into A498 and 786-O cell lines. The Cell Counting Kit-8 (CCK-8) assay and colony formation assay were used to observe the changes in cell proliferation after transfection. Flow cytometry was used to detect the effect on the cell cycle. Western blot was conducted to study the changes of related functional proteins.

**Results:**

The expression of TSG101 was higher in RCC tissues than in adjacent normal tissues. The CCK-8 assay showed that the proliferation and colony formation of the A498 and 786-O cell lines were attenuated after suppression of TSG101. Flow cytometry showed that silencing of TSG101 induced G0/G1 arrest. The western blot results revealed that the levels of cell cycle-related proteins (c-myc, cyclin E1 and cyclin-dependent kinase 2 (CDK2)) were markedly decreased in the siRNA groups.

**Conclusions:**

TSG101 promotes proliferation of RCC cells. This positive effect on tumor growth involves activation of c-myc and cyclin E1/CDK2 and their effect on cell cycle distribution.

## Introduction

Renal cell carcinoma (RCC) is one of the most refractory cancers in the world and accounts for about 2 to 3% of adult malignancies [1]. Among RCCs, clear cell RCC (ccRCC) is the most common subtype [2]. In addition to the undetermined pathogenesis, the nonspecific symptoms and metastatic lesions at initial diagnosis result in poor prognosis [3]. Currently, therapies targeting the vascular endothelial growth factor (VEGF) receptors and mammalian target of rapamycin (mTOR) are commonly applied in RCC treatment and have obtained a certain curative effect [4], yet some patients still do not achieve the expected efficacy due to drug resistance. Hence, surgery remains the main treatment of RCC [5, 6] and seeking novel therapeutic strategies and prognostic markers is critical.

The tumor susceptibility gene 101 (TSG101) is located in chromosome 11p15 and encodes a 46 kDa protein of 390 amino acid residues [7]. TSG101 is a multi-functional protein whose functions include the sorting and transport of endosomes [8–10], modulation of protein ubiquitination [11], and participation in p53/MDM2 feedback control loops [12, 13], thereby affecting epithelial cell growth and differentiation [14] and regulation of the cell cycle [15], with significant roles in the maintenance of cell homeostasis. To date, a growing body of evidence has indicated that TSG101 is overexpressed in various tumors [16–20], suggesting that TSG101 contributes to the promotion of cancers. Hence, we specifically down-regulated TSG101 using a small interfering RNA (siRNA) in order to observe its impact on the proliferation and cell cycle of RCC cells.

In this study, TSG101 was proved to be a novel oncogenic gene that facilitated RCC cell cycle progression. In addition, we predicted and verified that the effect of TSG101 on the growth of tumor was related to elevated c-myc protein levels, accompanied by up-regulated cyclin E1/cyclin-dependent kinase 2 (CDK2) complex. These findings may shed some light on the oncogenesis of RCC and provide more valuable strategies for the treatment of patients with RCC.

### Materials and methods

#### Clinical samples

A total of 15 paired tumor tissues were harvested from patients who received partial or radical nephrectomy at the Department of Urology of Shanghai Tenth People’s Hospital. The specimens were freshly frozen in liquid nitrogen until use. Informed consent was obtained from the patients and the study was approved by the ethics committee of the Tenth People’s Hospital of Tongji University (approved on February 23, 2017; approval # SHSY-IEC-KY -4.0/17–86/01), according to the tenets of the Declaration of Helsinki.

#### Immunohistochemistry

Fresh tissue samples were fixed in 4% paraformaldehyde, dehydrated through a graded series of ethanol solution and embedded in paraffin. Then the sections were deparaffinized in xylene and dehydrated with an ethanol gradient followed by blocking of endogenous peroxidase activity with 0.3% hydrogen peroxide in methanol for 20 min. Nonspecific binding was blocked by incubating the sections with 10% normal goat serum in phosphate buffered saline (PBS) for 1 h at room temperature. The sections were incubated with the primary antibody against TSG101 (1500, ab125011, Abcam, Cambridge, MA, USA) in PBS at 4 °C overnight. Horseradish peroxidase-polymer conjugated anti-rabbit IgG was used as the secondary antibody. The slides were stained with hematoxylin for visualization.

#### Cell culture

The human RCC cell lines A498 and 786-O were obtained from the American Type Culture Collection (ATCC, Manassas, VA, USA) and cultured in Dulbecco’s modified Eagle’s medium (DMEM) and RPMI-1640 (GIBCO, Life Technologies, Grand Island, NY, USA), respectively, supplemented with 10% fetal bovine serum (FBS; GIBCO) and 1% penicillin-streptomycin (Hyclone, Logan, Utah, USA), in an incubator at 37 °C with 5% CO_2_.

#### Transfection assay

A498 and 786-O cells (10^5^/well) were cultured in a 6-well plate before transfection. When the confluence reached 30–50%, transfection of the TSG101 siRNA and negative control (NC) siRNA was performed using the Lipofectamine 2000 Transfection kit (Invitrogen, Thermo Fisher Scientific, Inc., USA), according to the manufacturer’s instructions. The amount of siRNAs was 0.1 nmol. After 4–6 h of incubation, the medium was replaced with Opti-MEM (GIBCO, Life Technologies, Grand Island, NY, USA). The cells were used for future analysis 48 h after transfection.

The TSG101 and NC siRNAs were synthesized by Sangon Biotech Co., Ltd. (Shanghai, China). The sequence of the TSG101 siRNA was 5′-GCC UAC UAG UUC AAU GAC UTT-3′ (sense) and 5′-AGU CAU UGA ACU AGU AGG CTT-3′ (antisense), while the sequence of the NC siRNA was 5′-UUC UCC GAA CGU GUC ACG UTT-3′ (sense) and 5’-ACG UGA CAC GUU CGG AGA ATT-3′ (antisense).

#### Cell counting Kit-8 (CCK-8) and colony formation assay

The CCK-8 assay (Dojindo Molecular Technologies, Kimamoto, Japan) was used to assess cell proliferation, following the manufacturer’s instructions. Transfected cells were seeded into 96-well plates at 10^3^ cells/well. The CCK-8 reagent (10 μl) was added to the wells after 24 h and incubated for 2 h, followed by the measurement of optical density (OD) at 450 nm using a microplate spectrophotometer (Bio-Tek, Winooski, VT, USA). The transfected cells were seeded into 6-well plates at 10^3^ cells/well and used to perform the colony formation assay. After 8–10 days, the cells were washed with PBS lightly, fixed in 95% ethanol, and stained with 0.1% crystal violet.

#### Cell cycle analysis

The transfected cells were detached by EDTA-free trypsin (GIBCO), washed with precooled PBS, and fixed in 75% ethanol at 4 °C overnight. The cells were resuspended in 0.2 mL of PI/RNase Staining Buffer (BD Biosciences, San Jose, CA, USA) and incubated in the dark for 30 min. The cells were analyzed using a flow cytometer (BD Biosciences).

#### RNA extraction and reverse transcription-quantitative polymerase chain reaction (RT-qPCR)

According to the manufacturer’s protocol, total cellular RNA was extracted using TRIzol (Invitrogen) and stored at − 80 °C. For TSG101 detection, the cDNA was generated using the PrimeScript RT-PCR kit (Takara Bio, Inc., Otsu, Japan), following the manufacturer’s instructions. The RT conditions were 37 °C for 15 min, then 85 °C for 5 s. The SYBR-Green PCR master mix (Takara) was used for qPCR on a 7900HT Fast RT-PCR instrument (Applied Biosystems; Thermo Fisher Scientific). The amplification protocol was: 3 min at 95 °C, followed by 40 cycles at 95 °C for 3 s and 60 °C for 30 s. The TSG101 mRNA levels were normalized to the β-Actin mRNA levels using the 2^-ΔΔCt^ method. The primer sequences were: TSG101: 5′-GCC ACC TCT AGA ATG GCG GTG TCG GAG AGC C-3′ (F) and 5′-GGT GGC GTC GAC TCA GTA GAG GTC ACT GAG ACC-3′ (R); β-actin 5′-CAG AGC CTC GCC TTT GCC-3′ (F) and 5′-GTC GCC CAC ATA GGA ATC-3′ (R).

#### Western blot assay

Cells were washed with PBS twice and lysed in radioimmunoprecipitation assay lysis buffer (70 μL/well, Beyotime, Jiangsu, China) after 48–72 h of transfection. Protein concentration was determined using the bicinchoninic acid protein assay (Beyotime). Equal amounts of protein (25–50 μg) were loaded in 8% or 10% SDS-PAGE gel for electrophoresis. The proteins were transferred to nitrocellulose membranes (Sangon Biotech, Shanghai, China). The membranes were blocked at room temperature in 5% skimmed milk diluted with PBS plus Tween 20 (PBST) for 1 h and hybridized with the primary antibodies overnight at 4 °C. The membranes were washed with PBST three times and incubated with the appropriate secondary antibodies for 1 h. The membranes were washed with PBST three times and the protein bands were visualized using an Odyssey Scanner (LI-COR Biosciences, Lincoln, NE, USA).

The antibodies were: GAPDH (1:10,000, ab181602, Abcam, Cambridge, MA, USA), TSG01 (1:2000, ab125011, Abcam), c-myc (1:1000, 13,987, Cell Signaling, Danvers, MA, USA), cyclin E1 (1:1000, ab33911, Abcam), and CDK2 (1:1000, ab32147, Abcam).

#### Statistical analysis

Data were analyzed using SPSS 20.0 (IBM, Armonk, NY, USA) and GraphPad Prism software 5.0 (GraphPad Prism Software Inc., San Diego, CA, USA). All data were from at least three independent experiments and are presented as means ± standard deviation (SD). Student’s t-test was used for comparison and *p* < 0.05 was considered to be statistically significant.

## Results

### Expression of TSG101 was higher in RCC tumor tissues compared with adjacent normal tissues

**Fig. 1:**
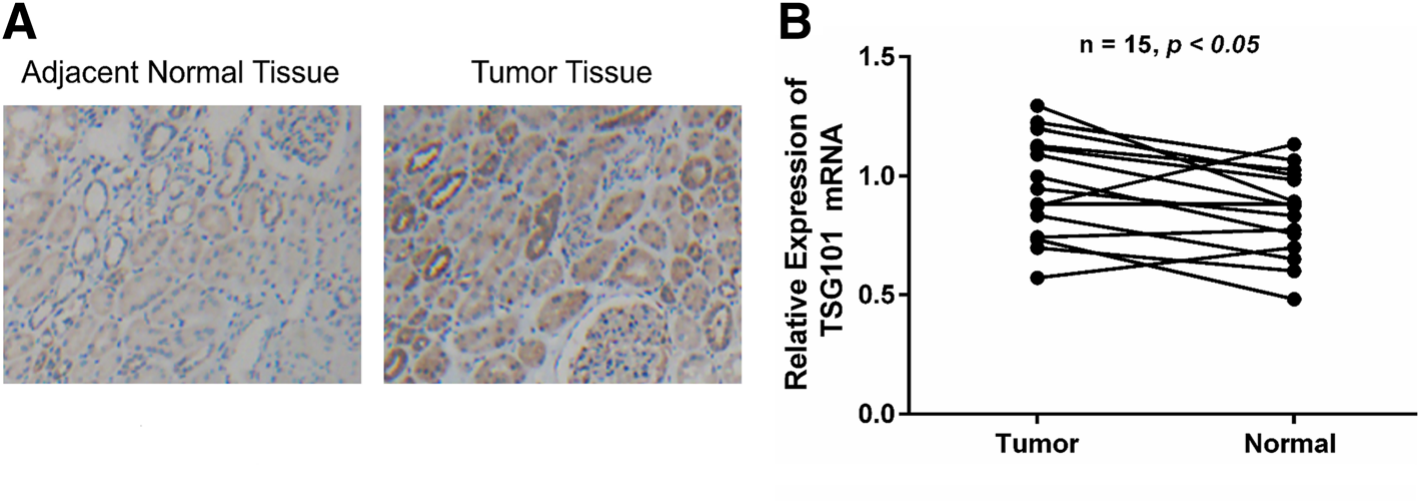
**a** Representative immunohistochemistry of TSG101 in tumor tissues compared to adjacent normal tissue of RCC patients (magnification × 100). **b** The expression of TSG101 mRNA was determined in tumor tissues and their matched adjacent normal tissues by qRT-PCR

Previous studies have identified TSG101 as an oncogene in various tumor types [19–21]. First, we detected the TSG101 expression in RCC tissues compared with in paracancerous normal tissues. The protein level of TSG101 was examined by immunohistochemistry. Representative staining of TSG101 in tumor tissues compared to adjacent normal tissue of RCC patients is shown in Fig. 1a. As expected, compared with adjacent normal tissues, TSG101 mRNA expression was higher in RCC tumor tissues (*p* < 0.05) (Fig. 1b). These data hint that high expression of TSG101 is associated with RCC development.

### Expression of TSG101 was obviously reduced by siRNA

**Fig. 2:**
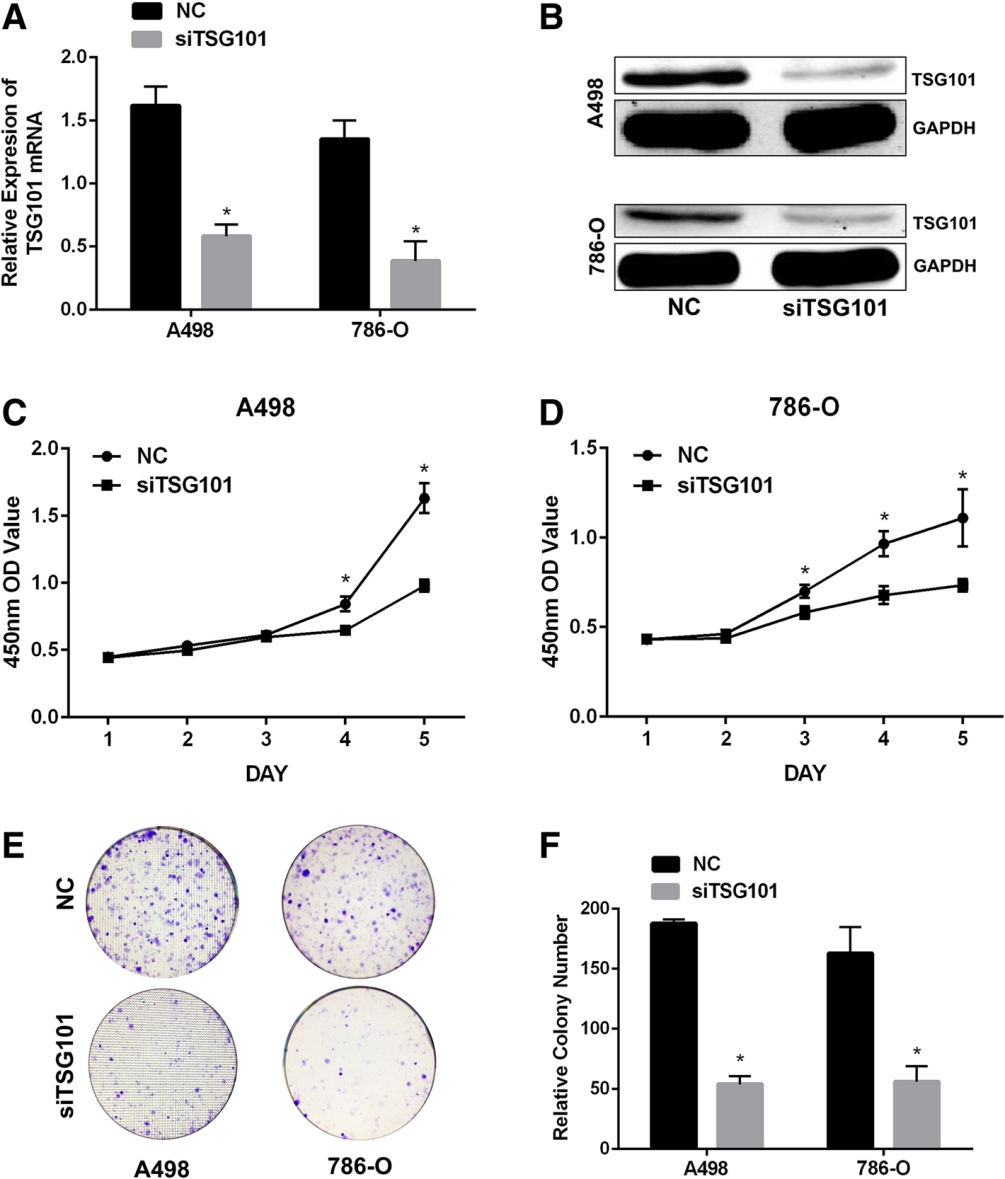
Knockdown of TSG101 inhibited cell proliferation and colony formation ability in RCC. **a** qRT-PCR and (**b**) western blot confirmed that TSG101 could be silenced successfully by siRNA. CCK-8 assays of (**c**) A498 and (**d**) 786-O cells transfected with siTSG101 or NC. **e** and **f** Colony formation assays of A498 and 786-O cells transfected with siTSG101 or NC. Representative images and average colony numbers are shown. Data are presented as means ± SD. * *p* < 0.05 vs. NC (*n* = 3)

To further investigate the potential function of TSG101, the RCC cell lines A498 and 786-O were selected for the RNA interference experiment and transfected with siRNA targeting human TSG101 (siTSG101) and a NC siRNA. The silencing efficiency of the siRNA on TSG101 expression was detected by western blot and qRT-PCR (*p* < 0.05) (Fig. 2a and b). The data showed that the siRNA exclusively targeted TSG101. Compared with NC siRNA, siTSG101 reduced endogenous TSG101 expression in A498 and 786-O cells.

### TSG101 downregulation inhibited cell proliferation in RCC

We performed CCK-8 and colony formation assays to examine the effect of TSG101 on the proliferation of RCC cells. As determined by the CCK-8 assay, silencing TSG101 slowed cell proliferation in a time-dependent manner in A498 and 786-O cells compared with the cells transfected with NC siRNA (*p* < 0.05) (Fig. 2c and d). The results of the colony formation assay supported those of the CCK-8 assay (*p* < 0.05) (Fig. 2e and f). These results, taken together, indicate that TSG101 could enhance RCC cell proliferation.

### Knockdown of TSG101 led to RCC cell cycle arrest

**Fig. 3:**
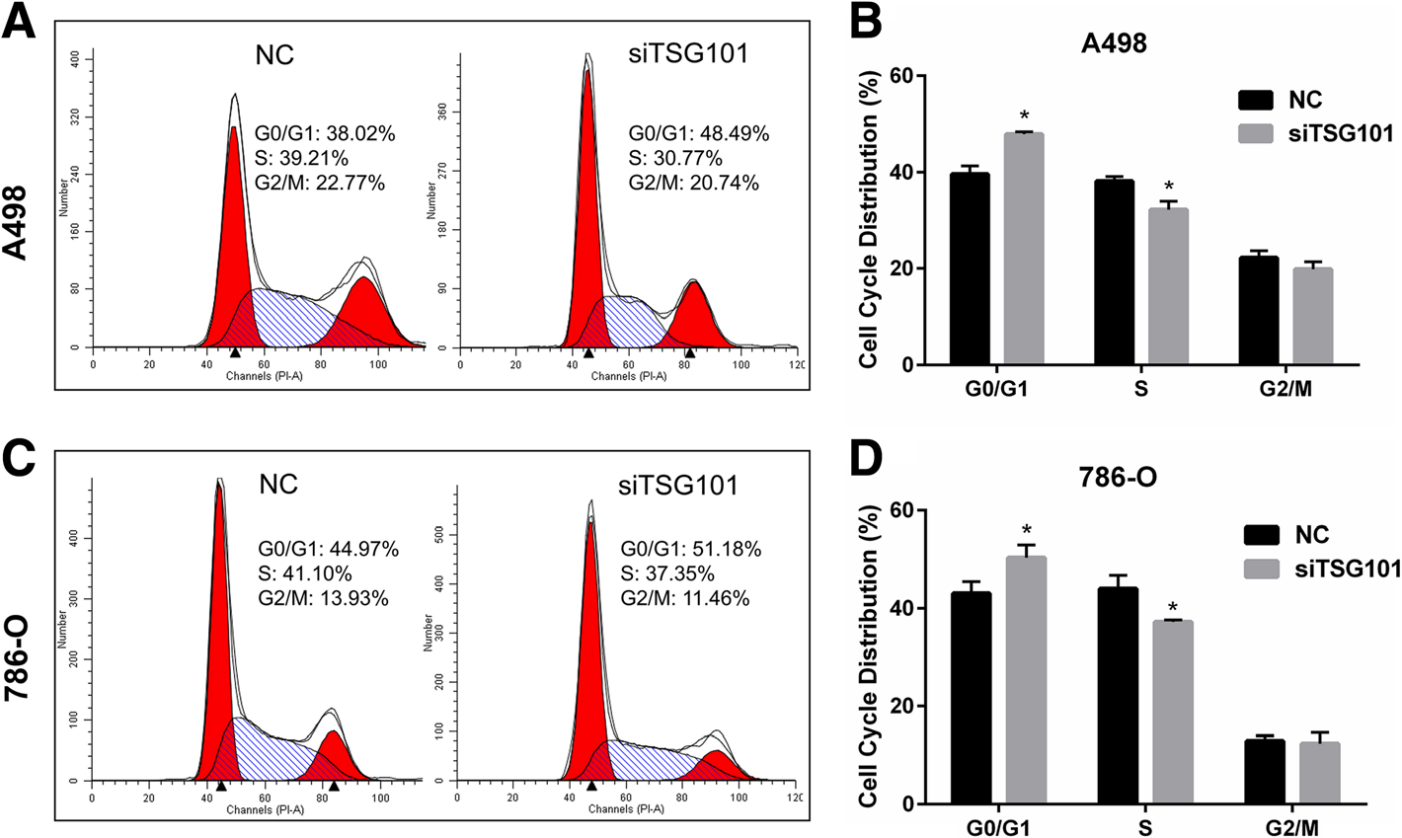
TSG101 downregulation induced RCC cell cycle arrest. Flow cytometry in (**a**) A498 and (**c**) 786-O cells transfected with siTSG101 or NC. **b** and **d** The G0/G1, S, and G2/M phase proportions of A498 and 786-O cells transfected with siTSG101 or NC. Data are presented as means ± SD. * *p* < 0.05 vs. control group (n = 3)

Cell cycle analysis was conducted to investigate the mechanisms by which the silencing of TSG101 blunted cell proliferation. The cell cycle distribution was altered by the inhibition of TSG101. The proportion of G0/G1 phase cells was increased and the proportion of S phase cells was reduced by down-regulated TSG101 in A498 and 786-O cell lines (*p* < 0.05) (Fig. 3). Based on these data, we hypothesized that downregulation of TSG101 may inhibit proliferation by inducing cell cycle arrest in RCC cells.

### Cell cycle-related proteins levels were suppressed by inhibition of TSG101 in RCC cells

**Fig. 4:**
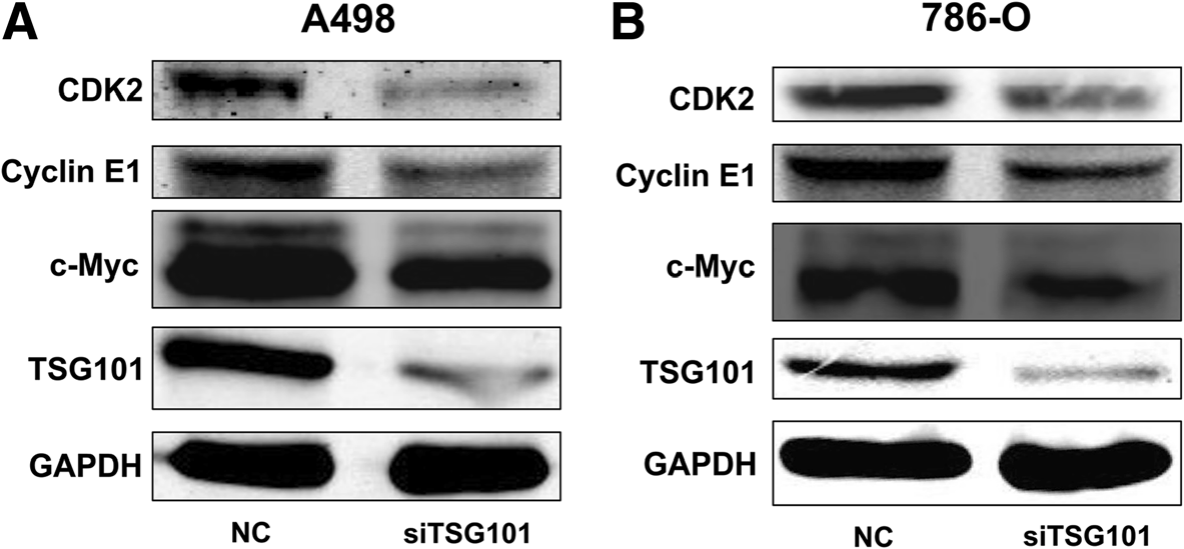
Cell cycle related proteins were suppressed by inhibition of TSG101 in RCC cells. Western blot analysis of c-myc, CDK2 cyclin E1, and TSG101 protein levels of the siTSG101 group compared with the NC group in (**a**) A498 and (**b**) 786-O cells

Cyclins are a family of proteins that control the progression of the cell cycle by activating CDK enzymes and their aberrant expression disturbs the cell cycle [22, 23]. In addition, inhibition of c-myc in growing cells leads to G1 arrest with a consequent decrease of cyclin E and CDK2 [24]. As shown in Fig. 4, western blotting showed that compared with those in the NC groups, the expression levels of cell cycle-associated proteins (c-myc, cyclin E1, and CDK2) were markedly reduced in the siRNA group, supporting the results of the cell cycle analysis. These results implied that TSG101 regulated, at least in part, the cell cycle of RCC cells through cyclin E1/CDK2 activity, which has a positive correlation with c-Myc.

## Discussion

Since the study by Li et al. that reported TSG101 as a novel tumor susceptibility gene [25], a number of studies have demonstrated that high expression of TSG101 in numerous malignant tumors was responsible for cancer cell growth. An early study verified that the upregulation of TSG101 might boost carcinogenesis in papillary thyroid carcinoma [21], followed by a study that proved that its expression might be necessary for processes involved in tumor progression [20]. Moreover, recent studies also showed that positive TSG101 expression was associated with the clinical, pathological, and biological behaviors and with poor prognosis in breast cancer and hepatocellular carcinoma [18, 19]. These findings highlight TSG101 as a cancer-promoting gene, but until now, no report has investigated the role of TSG101 in the development of RCC.

In the present study, siRNA was used to down-regulate endogenous TSG101 to study the role of TSG101 in RCC cell lines and thereby explored the association between TSG101 and tumorigenesis of RCC. The CCK-8 and colony formation assays revealed that depletion of TSG101 reduced cell growth, which was in accordance with previous studies. Furthermore, inhibition of TSG101 promoted RCC cells to be arrested in the G0/G1 phase.

The accurate transition from G1 phase to S phase is crucial for the control of eukaryotic cell proliferation. Cyclin E1/CDK2 kinase activity peaks at the G1/S phase and is required for proper cell cycle progression into the S phase [26, 27]. Beyond question, the dysregulation of cyclin E1/CDK2 kinase activity is involved in oncogenesis in ovarian cancer [28], bladder cancer [29], and breast cancer [30], among others. C-myc also regulates the cell cycle and contributes to the formation of cancers [31–33]. In addition, a previous study constructed a mutant c-myc protein (named MadMyc) that could cause repression of the c-myc target genes; the study showed that the MadMyc-induced G1 arrest was rescued by ectopic expression of cyclin E and CDK2 [24]. So far, several studies have reported that c-myc had a bearing on cyclin E1/CDK2 kinase activity [34–36]. Interestingly, this same situation could be observed in the present study. Indeed, repression of TSG101 reduced the protein levels of c-myc, cyclin E1, and CDK2 in RCC cells.

In conclusion, the present study used siRNA to provide some evidence that TSG101 has a positive influence on RCC cell proliferation and that this effect may rely on proteins that are involved in cell cycle regulation, including c-myc, cyclin E1, and CDK2. These findings might help explore the terra incognita of RCC tumorigenesis and could provide promising strategies for the treatment of RCC.

## Abbreviations

CCK-8: Cell Counting Kit-8
CDK2: cyclin-dependent kinase 2
mTOR: mammalian target of rapamycin
PBS: phosphate buffered saline
RCC: renal cell carcinoma
siRNA: small interfering RNA
TSG101: tumor susceptibility gene 101
VEGF: vascular endothelial growth factor
